# Motion monitoring during a course of lung radiotherapy with anchored electromagnetic transponders: Quantification of inter- and intrafraction motion and variability of relative transponder positions

**DOI:** 10.1007/s00066-017-1183-0

**Published:** 2017-07-21

**Authors:** Daniela Schmitt, Simeon Nill, Falk Roeder, Daniela Gompelmann, Felix Herth, Uwe Oelfke

**Affiliations:** 1 Division of Medical Physics in Radiation Oncology, German Cancer Research Center (DKFZ), Heidelberg, Germany; 2 Heidelberg Institute for Radiation Oncology (HIRO), National Center for Radiation Research in Oncology (NCRO), Heidelberg, Germany; 3 Department of Radiation Oncology, Heidelberg University Hospital, Im Neuenheimer Feld 400, 69120 Heidelberg, Germany; 4 Joint Department of Physics, The Institute of Cancer Research and The Royal Marsden NHS Foundation Trust, London, UK; 5 Clinical Cooperation Unit Molecular Radiooncology, German Cancer Research Center (DKFZ), Heidelberg, Germany; 6 Department of Radiation Oncology, University of Munich (LMU), Munich, Germany; 7 Pneumology and Critical Care Medicine, Thoraxklinik, University of Heidelberg, Heidelberg, Germany; 8 Translational Lung Research Center Heidelberg (TLRC), German Center for Lung Research, Heidelberg, Germany

**Keywords:** Intrafraction motion, Lung radiotherapy, Electromagnetic tracking, Stability of marker positions, Motion management, Intrafraktionelle Bewegung, Strahlentherapie der Lunge, Elektromagnetisches Tracking, Stabilität von Markerpositionen, Bewegungsmanagement

## Abstract

**Purpose:**

Anchored electromagnetic transponders for tumor motion monitoring during lung radiotherapy were clinically evaluated. First, intrafractional motion patterns were analyzed as well as their interfractional variations. Second, intra- and interfractional changes of the geometric transponder positions were investigated.

**Materials and methods:**

Intrafractional motion data from 7 patients with an upper or middle lobe tumor and three implanted transponders each was used to calculate breathing amplitudes, overall motion amount and motion midlines in three mutual perpendicular directions and three-dimensionally (3D) for 162 fractions. For 6 patients intra- and interfractional variations in transponder distances and in the size of the triangle defined by the transponder locations over the treatment course were determined.

**Results:**

Mean 3D values of all fractions were up to 4.0, 4.6 and 3.4 mm per patient for amplitude, overall motion amount and midline deviation, respectively. Intrafractional transponder distances varied with standard deviations up to 3.2 mm, while a maximal triangle shrinkage of 36.5% over 39 days was observed.

**Conclusions:**

Electromagnetic real-time motion monitoring was feasible for all patients. Detected respiratory motion was on average modest in this small cohort without lower lobe tumors, but changes in motion midline were of the same size as the amplitudes and greater midline motion can be observed in some fractions. Intra- and interfractional variations of the geometric transponder positions can be large, so for reliable motion management correlation between transponder and tumor motion needs to be evaluated per patient.

**Electronic supplementary material:**

The online version of this article (doi: 10.1007/s00066-017-1183-0) contains supplementary material, which is available to authorized users.

## Zusammenfassung

**Ziel:**

Verankerte, elektromagnetische Transponder für die Bewegungserkennung des Tumors während der Strahlentherapie der Lunge wurden klinisch evaluiert. Dafür wurden intrafraktionelle Bewegungsmuster und ihre interfraktionellen Variationen analysiert und intra- und interfraktionelle Veränderungen der geometrischen Transponderpositionen untersucht.

**Material und Methoden:**

Intrafraktionelle Bewegungsdaten von 7 Patienten mit einem Ober- oder Mittellappentumor und je drei implantierten Transpondern wurden benutzt, um Atemamplituden, Gesamtbewegungsaufkommen und Bewegungsmittellinien für die drei zueinander senkrechten Bewegungsrichtungen und dreidimensional (3D) in 162 Fraktionen zu berechnen. Für 6 Patienten wurden intra- und interfraktionelle Variationen der Transponderabstände und der Größe des von den Transponderpositionen definierten Dreiecks im Therapieverlauf bestimmt.

**Ergebnisse:**

Die mittleren 3D-Werte aller Fraktionen pro Patient ergaben bis zu 4,0, 4,6 und 3,4 mm für Amplitude, Gesamtbewegungsaufkommen und Mittellinienveränderung. Intrafraktionelle Transponderabstände variierten mit Standardabweichungen von bis zu 3,2 mm, während eine maximale Verkleinerung der Dreiecksgröße um 36,5 % über 39 Tage beobachtet wurde.

**Schlussfolgerung:**

Elektromagnetische Echtzeit-Bewegungserkennung war für alle Patienten durchführbar. In dieser kleinen Patientenkohorte ohne Unterlappentumore war die detektierte Atembewegung im Mittel moderat. Jedoch waren Veränderungen der Bewegungsmittellinie in der gleichen Größe wie die Atemamplituden; in einzelnen Fraktionen konnten größere Mittellinienbewegungen beobachtet werden. Intra- und interfraktionelle Variationen der geometrischen Transponderpositionen können erheblich sein, so dass für ein zuverlässiges Bewegungsmanagement die Korrelation zwischen Transponder- und Tumorbewegung für jeden Patienten untersucht werden sollte.

Radiotherapy is a cornerstone of curative intent treatment for early and advanced lung cancer, see e. g. [1–6]. Thereby intrafractional tumor motion is a main challenge because it potentially causes deviations between the planned and delivered doses in high precision therapy [7]. A new method for three-dimensional (3D) real-time lung tumor motion monitoring without imaging dose is the continuous detection of implanted anchored electromagnetic transponders [8] available for the Calypso® system (Varian Medical Systems, Inc., Palo Alto, CA, USA). These transponders have a capsule with expandable nitinol legs for fixation in small airways and are implanted in lung tissue bronchoscopically under continuous fluoroscopic guidance within 3 cm distance to the lesion. Recently, a report of the first patient treated with multileaf collimator (MLC) tracking based on them was published [9]. Here we report the evaluation of collected motion data from patients studied at our center who have been enrolled in a multicenter trial investigating safety and stability of the anchored transponders in a wider patient collective. Each patient had three transponders implanted and the Calypso® system reported single transponder as well as transponder-centroid translations in lateral, longitudinal and vertical direction. Each transponder was updated with a rate of approximately 3.3 Hz, leading to a continuously updated centroid position of approximately 10 Hz, always using the latest position information of each transponder. With this data we were able to quantify the intrafractional motion during lung treatments and their interfractional variations. Additionally, we evaluated the stability of intrafractional motion within fractions because all systems which aim to adapt treatment directly after motion detection (e. g. MLC tracking [10–12]) need motion prediction due to system latency, see e. g. [13, 14]. The quality of these predictions depend on intrafractional regularity of the motion patterns. To study this regularity, motion patterns of the two patients with the largest 3D amplitudes were further analyzed via quantification of changes between adjacent beam-on and beam-off phases. Since the transponder-centroid position is only a surrogate for motion of the lung lesion, we quantified the intra- and interfractional changes of individual transponder positions via calculation of intertransponder distance variation.

## Materials and methods

### Patient data

Seven lung cancer patients suffering from an upper or middle lobe tumor were enrolled at the German Cancer Research Center within the aforementioned multicenter trial (ClinicalTrials.gov NCT01396551) organized by Varian Medical Systems, Inc. The trial was approved by the ethics committee of the medical faculty at Heidelberg university. Intrafractional motion data of the bronchoscopically implanted electromagnetic transponders was used for all presented investigations. Two patients received hypofractionated 3D conformal radiotherapy (3D CRT) in 10 fractions and 5 patients were treated with step-and-shoot IMRT in 28–31 fractions. Tumor positions and number of evaluated fractions for our study are listed in Table 1. For the evaluations of single transponder motions one IMRT patient (P5) was excluded because one transponder had to be omitted for motion monitoring as the distance of it to one of the other transponders exceeded the maximal allowed distance of 7.5 cm specified by the manufacturer [15]. Average delivery time of a treatment fraction was 4.5 min (9.6 min) for 3D CRT (IMRT) patients. For all investigations single transponder and transponder-centroid data were linearly interpolated to the same 10 Hz grid to obtain synchronized information for all transponders and to equalize the original fluctuations in update rate.

**Table 1:** Results of transponder-centroid motion evaluation for all patients

Patient		P1	P2	P3	P4	P5	P6	P7
Tumor position		RUL	LUL	LUL	RML	LUL	RML	RML
Evaluated fractions		10	10	31	26	29	29	27
Mean of fraction midline (mean ± SD) [mm]	Lat	0.0 ± 0.3	−0.2 ± 0.3	0.1 ± 0.4	0.4 ± 0.7	0.4 ± 0.5	0.1 ± 1.5	0.4 ± 0.5
	Long	0.7 ± 1.7	−0.1 ± 0.3	0.3 ± 0.5	0.7 ± 0.8	0.2 ± 0.5	−1.0 ± 1.4	0.4 ± 0.5
	Vert	−1.5 ± 1.6	0.1 ± 0.5	0.0 ± 0.4	0.1 ± 0.4	0.0 ± 1.1	−0.6 ± 1.3	0.0 ± 0.5
	3D^a^	3.4 ± 0.8	0.8 ± 0.3	0.8 ± 0.4	1.3 ± 0.7	1.4 ± 0.6	2.9 ± 1.0	1.0 ± 0.4
SD of fraction midline (mean ± SD) [mm]	Lat	0.4 ± 0.1	0.3 ± 0.1	0.3 ± 0.1	0.4 ± 0.2	0.4 ± 0.2	1.0 ± 0.5	0.4 ± 0.2
	Long	1.8 ± 0.4	0.3 ± 0.1	0.3 ± 0.3	0.6 ± 0.3	0.4 ± 0.1	1.2 ± 0.6	0.4 ± 0.2
	Vert	1.9 ± 0.9	0.3 ± 0.1	0.3 ± 0.1	0.3 ± 0.1	0.7 ± 0.2	0.9 ± 0.4	0.4 ± 0.1
	3D^b^	1.8 ± 0.5	0.3 ± 0.1	0.4 ± 0.3	0.6 ± 0.3	0.6 ± 0.2	1.2 ± 0.5	0.5 ± 0.2
Mean of overall fraction motion (mean ± SD) [mm]	Lat, long and vert have the same values as for “Mean of fraction midline”							
	3D	4.6 ± 0.8	1.5 ± 0.2	0.9 ± 0.4	2.8 ± 0.5	2.1 ± 0.6	3.4 ± 0.9	1.2 ± 0.3
SD of overall fraction motion (mean ± SD) [mm]	Lat	0.8 ± 0.1	0.9 ± 0.2	0.4 ± 0.1	1.3 ± 0.3	0.7 ± 0.2	1.6 ± 0.4	0.5 ± 0.2
	Long	3.1 ± 0.3	0.5 ± 0.1	0.4 ± 0.3	2.4 ± 0.3	0.6 ± 0.2	1.9 ± 0.5	0.7 ± 0.2
	Vert	3.0 ± 0.8	1.1 ± 0.2	0.4 ± 0.1	0.5 ± 0.1	1.8 ± 0.5	1.1 ± 0.4	0.6 ± 0.1
	3D	2.4 ± 0.4	0.6 ± 0.1	0.4 ± 0.3	1.3 ± 0.4	1.1 ± 0.3	1.5 ± 0.5	0.6 ± 0.2
Amplitude around current midline (mean ± SD) [mm]	Lat	0.9 ± 0.2	1.1 ± 0.3	0.4 ± 0.1	1.4 ± 0.3	0.8 ± 0.2	1.6 ± 0.3	0.3 ± 0.1
	Long	3.1 ± 0.6	0.5 ± 0.1	0.2 ± 0.0	3.2 ± 0.5	0.4 ± 0.2	2.1 ± 0.3	0.7 ± 0.2
	Vert	2.7 ± 0.3	1.6 ± 0.2	0.3 ± 0.0	0.5 ± 0.1	2.2 ± 0.7	0.7 ± 0.2	0.5 ± 0.1
	3D	4.0 ± 0.6	2.0 ± 0.3	0.5 ± 0.1	3.4 ± 0.5	2.3 ± 0.7	2.8 ± 0.3	0.9 ± 0.2

*RUL* right upper lobe, *LUL* left upper lobe, *RML* right middle lobe, *lat* lateral, *long* longitudinal, *vert* vertical, *SD* standard deviation, *3D* three dimensional

Referring to the supplementary material:

^a^ $$\mathrm{M}_{\text{mean}}^{\text{mean}}\pm \mathrm{M}_{\text{mean}}^{\text{SD}}$$

^b^ $$\mathrm{M}_{\text{SD}}^{\text{mean}}\pm \mathrm{M}_{\text{SD}}^{\text{SD}}$$

### Transponder-centroid motion quantification

The basis of all motion evaluations was the determination of a transponder-centroid motion midline by calculating a sliding mean for a time window of 10 s, covering 2–3 breathing cycles. The three-dimensional midline $\mathrm{\bf{M}}_{\mathrm{t}}^{\mathrm{f}}$ was calculated for each fraction f for all points in time t between the start of the first and the end of the last treatment beam, using detected positions $\mathrm{\bf{r}}_{\mathrm{t}}^{\mathrm{f}}$ including the 5 s before and after this time interval (equal to 50 data points before and after, because of the 10 Hz grid). All evaluations were made equivalently for the unidirectional components and the norm $\mathrm{M}_{\mathrm{t}}^{\mathrm{f}}$ of the vector, which is used for description in this section and the formulas in the supplementary material.

The midline position for the time the irradiation started $\mathrm{t}_{\mathrm{s}}$ was called initial midline: $\mathrm{M}_{\text{initial}}^{\mathrm{f}}=\mathrm{M}_{\mathrm{t}=\mathrm{t}_{\mathrm{s}}}^{\mathrm{f}}.$ The midline variations per fraction were calculated as mean $\mathrm{M}_{\text{mean}}^{\mathrm{f}}$ and standard deviation (SD) $\mathrm{M}_{\text{SD}}^{\mathrm{f}}$ of the midline positions relative to$\mathrm{M}_{\text{initial}}^{\mathrm{f}}$. From these fraction values means and SDs were calculated per patient to quantify the interfractional variations of the mean midline deviation ($\mathrm{M}_{\text{mean}}^{\text{mean}}$ and $\mathrm{M}_{\text{mean}}^{\text{SD}}$) and the interfractional variations of the intrafractional width of midline positions ($\mathrm{M}_{\text{SD}}^{\text{mean}}$ and $\mathrm{M}_{\text{SD}}^{\text{SD}}$).

For quantification of overall motion amount all centroid positions $\mathrm{\bf{r}}_{\mathrm{t}}^{\mathrm{f}}$ were analyzed in relation to the corresponding $\mathrm{\bf{M}}_{\text{initial}}^{\mathrm{f}}$ for all three directions separately and in 3D. For each fraction, means and SDs were calculated and from these values interfractional means and SDs per patient as above.

For each in- and exhale peak the distance between $\mathrm{\bf{r}}_{\mathrm{t}}^{\mathrm{f}}$ and the midline defined for the same point in time $\mathrm{\bf{M}}_{\mathrm{t}}^{\mathrm{f}}$ was calculated to determine motion amplitudes in lateral, longitudinal and vertical direction and in 3D relative to the actual midline. Data from all peaks were averaged to derive a mean fraction amplitude. From these amplitudes the mean amplitude per patient was calculated with the corresponding SD.

For investigation of intrafractional changes between adjacent beam-on and beam-off phases motion patterns of the two patients with the largest 3D amplitudes were further analyzed. For each beam-off time (lasting about 20 s), three intervals of the same length I_1_, I_2_ and I_3_ were defined, where I_2_ refers to the beam-off interval and I_1_ and I_3_ denote the adjacent beam phases before and after the beam-off time. For these intervals the SD of centroid positions, as surrogate for overall motion amount and the mean motion midline, were determined for three mutually perpendicular directions and in 3D. The differences between these adjacent intervals were evaluated.

**Fig. 1:**
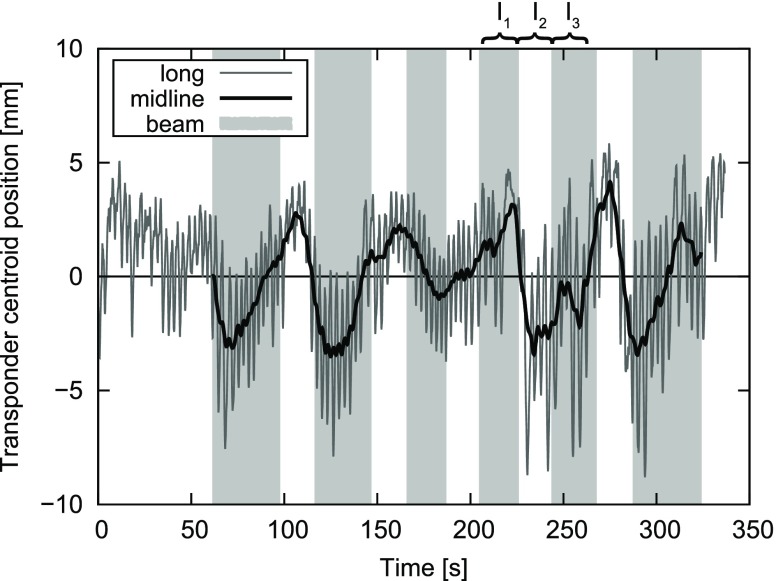
Example of longitudinal transponder-centroid motion for fraction 4 of patient 1. The longitudinal midline and the beam-on times are also indicated. The initial midline is set to zero. The intervals I_1_, I_2_ and I_3_ related to the 4th beam-off phase are shown as example.

An example of detected motion with plotted midline and indicated times of irradiation can be found in Fig. 1.

### Intertransponder variations

Variations in transponder positions were evaluated regarding intra- and interfractional distance changes. First, transponder distances were calculated from the interpolated single transponder data for all points in time between start of first and end of last beam for all three directions. From these distances the fraction SDs were calculated.

To study the origin of intrafractional variations, linear correlations between the longitudinal SDs of transponder distances and three parameters potentially affecting relative transponder motion were evaluated. These parameters were (I) the fraction mean 3D distance of the transponder pair, (II) the mean 3D amplitude of the transponder-centroid in the corresponding fraction and (III) the product of (I) and (II).

In addition, the interfractional changes in the individual transponder positions were analyzed. Therefore 3D transponder distances were used for determination of the area of the triangle defined by the location of the transponders. For each fraction, the mean and SD of the triangle area were calculated to evaluate the overall trend. Linear regressions of the 3D distances with the day after first fraction were averaged to determine a mean distance change per day (6 patients × 3 transponder distances = 18 regressions). Furthermore, mean 3D transponder distances of the first fraction were defined as patient-specific initial distances and it was investigated which percentage of the 381 mean distances measured in the following fractions were within 1 and 2 mm.

## Results

### Transponder-centroid motion quantification

**Fig. 2:**
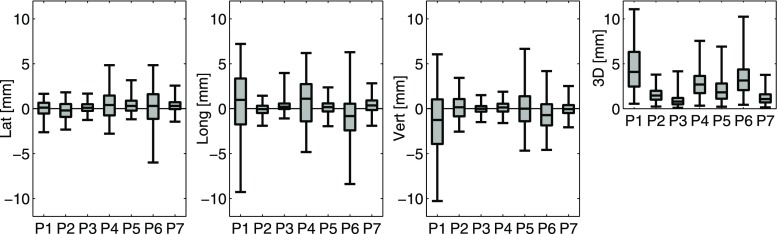
Transponder-centroid displacements for all patients in three directions and in 3D calculated as deviations from the initial midline for all fractions (overall motion amount). The box indicates the values between 25 and 75% of the motion data and the vertical line within the box marks the median. To exclude outliers, the whiskers cover only 99% of the data. *Lat* lateral, *Long* longitudinal, *Vert* vertical, *3D* three dimensionally

The overall motion amount for all patients can be seen in Fig. 2 as a boxplot of all transponder-centroid positions relative to the corresponding initial midline. Quantitative results of the intra- and interfractional changes in motion midline and overall motion are presented in Table 1 with the breathing amplitudes. The unidirectional motion midline means of fraction means range from −1.5 to 0.7 mm with SDs between 0.3 and 1.6 mm representing the interfractional variations. The majority of these means of fraction means are very close to zero as anticipated for a periodic breathing motion. The corresponding calculations for the mean of overall motion resulted in the same values, which has to be expected, as the midline mean is calculated from nearly the same data (plus 5 s in the beginning and the end of each fraction) using one additional averaging. The three-dimensional means of fraction means, whose results are not dominated by the compensation of positive and negative position values, show values up to 3.4 mm for the motion midline and up to 4.6 mm for the overall motion. These values are not the same because of the additional calculation of vector norm, which does not commute with the averaging. The means and SDs of the fraction SDs in midline and overall motion amount describe the intrafractional variations and their change between fractions. Because of the averaging, means of SDs are generally larger for the overall motion than for the midline. Maximum unidirectional values are 3.0 and 1.9 mm, respectively. Maximum SD describing interfractional changes is 0.9 mm.

For two of the seven patients no amplitude was observed to be larger than 1 mm. The maximum unidirectional amplitudes of the other five patients are either in the longitudinal or the vertical direction, ranging from 1.6 to 3.2 mm. Three-dimensional amplitudes were observed to be between 0.5 and 4.0 mm, while the interfractional change of amplitudes was small with all SDs well below 1 mm.

The two patients with the largest 3D amplitude are P1 and P4. The evaluation of motion differences in adjacent beam-on and beam-off phases considers 3 intervals for each of the 5 beam-off times per fraction for P1 and 8 beam-off times per fraction for P4 (all IMRT segments with the same gantry angle are summarized to one beam including the short breaks between segments). P1 shows remarkable deviations between adjacent intervals, e. g. a mean difference of vertical midline of 1.4 mm and maximum values of 7.0 mm for longitudinal and 9.1 mm for vertical midline, while for P4 (second largest motion amount) all studied differences are negligible.

### Intertransponder variations

**Fig. 3:**
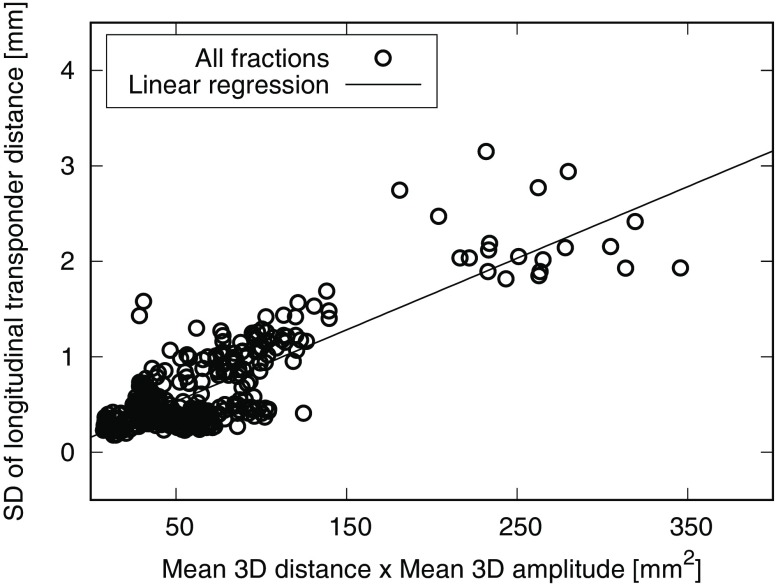
Standard deviations (*SD*) of longitudinal transponder distances plotted against the product of the mean three-dimensional (*3D*) transponder distance and the mean 3D amplitude of the transponder-centroid for each transponder pair in each fraction of all 6 patients

Intrafractional longitudinal transponder distances vary with SDs up to 3.2 mm. All lateral and vertical SDs are below 1 mm, hence they were not studied further. Correlation analyses between longitudinal distance SDs with 3D transponder distances (Pearson’s correlation coefficient r = 0.42) and 3D transponder-centroid amplitudes (r = 0.49) show poor results, while their product is strongly correlated to the longitudinal SDs (r = 0.83, *p* < 0.001). This shows that only the coincidence of large transponder distances and overall motion amount leads to large intrafractional transponder distance variations. Fig. 3 shows the plot of all longitudinal SDs of all patients against the corresponding product of mean 3D transponder distance and mean 3D transponder-centroid amplitude per fraction.

**Fig. 4:**
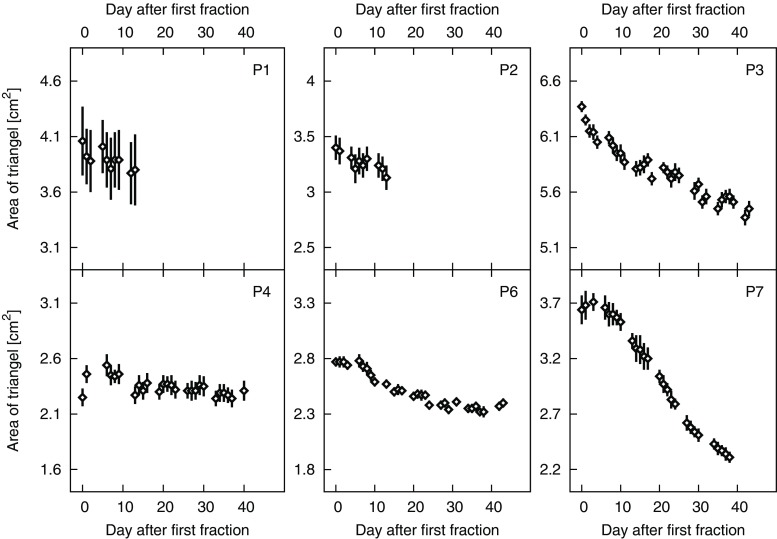
Triangle area defined by the transponder positions per day of all 6 patients (means with standard deviations depicted by error bars). Each vertical axis covers a range of 2 cm^2^

For all 6 patients the transponder triangle area shrank over the treatment course, see Fig. 4. A maximum decrease of 36.5% was observed for P7. The triangle shrinkage resulted from heterogeneous interfractional transponder distance variations; 16 of 18 distances shrank and 2 distances grew. The mean 3D transponder distance change per day after first fraction, determined via averaging of regressions, resulted in −0.07 mm ± 0.08 mm. The largest slope in the linear regression, namely −0.25 mm per day was found in P7. Considering all mean 3D transponder distances per fraction, the difference to the first fraction is maximum 1 mm (2 mm) for 47% (73%) of distances.

## Discussion

### Transponder-centroid motion quantification

Published analyses of lung tumor motion found an increasing motion amount with decreasing distance to the diaphragm, e. g. [16]. Because the tumors in the current work are located in an upper or middle lobe, data from lower lobe tumors were removed from published data for comparison, if not stated different. In the current study, amplitude is defined as distance from midline to peak, while in the image-based data, used for comparison in the following, a peak-to-peak amplitude is used. Therefore, current results were doubled for comparison finding good agreement between image-based and electromagnetically detected motion. Considering 11 patients from an analysis using respiration correlated cone-beam CT (CBCT) [17], mean 3D amplitude extracted from 6 CTs per patient can be calculated to 3.7 mm ± 3.2 mm. The corresponding value for our data is 4.5 mm ± 2.5 mm. Mean unidirectional amplitudes determined from lung marker motion detected fluoroscopically with 30 Hz [16] result in 1.2 mm ± 0.9 mm, 3.2 mm ± 3.2 mm and 2.6 mm ± 2.2 mm for lateral, longitudinal and vertical motion, respectively, considering only the 13 patients with tumors in an upper or middle lobe. Our data results in 1.8 mm ± 1.0 mm, 2.9 mm ± 2.6 mm and 2.4 mm ± 1.9 mm.

Large intrafractional changes in motion midline were observed, especially in P1, where the mean of midline deviations from fraction specific initial midline over all fractions is 3.4 mm in 3D. The largest difference in unidirectional mean midline position between two consecutive intervals of approximately 20 s was found in the same patient to be 9.1 mm in vertical direction. Maximum longitudinal drift in mean tumor position determined from repeated 4D CT scans [18] was 6.9 mm for one lower lobe tumor within 30 min.

These results show the possibility of relevant variations in motion patterns during a course of treatment. This should be kept in mind during margin definition for conventional therapy without continuous motion detection based on one pretreatment 4D CT as well as for beam-tracking techniques relying on aforementioned motion prediction methods.

### Intertransponder variations

The amount of intrafractional variation in transponder distances found here depends on the combination of intertransponder distance and overall motion amount. As the motion amount for each patient is unknown before therapy, the intertransponder distances should be as small as possible, regarding minimum distance of 1 cm requested by the system [9]. Concurrently, for good representation of tumor motion it is desirable to enclose the whole tumor with the transponders, while the transponder-centroid should be as close to the tumor-centroid as possible. Since there is no published data available for intrafractional distance variations of implanted lung markers, only interfractional variations in 3D distance found here can be compared with published data on bronchoscopically placed gold markers [19]. Reported intermarker distances measured with stereoscopic fluoroscopic x‑ray imaging for 11 hypofractionated patients during 1 to 2 weeks of treatment differ maximum 1 mm (2 mm) from the initial distances (determined from planning CT) for 80% (95%) of 198 measurements in 71 setups. The fraction mean 3D transponder distances in this work differs much more from the first fraction with 47% (73%) for 1 mm (2 mm). But, considering only the first 14 days after start of treatment, our values increase to 71% (88%) and for the first 7 days, they grow to 81% (90%). Thus, the transponder or marker distance changes seem not to arise from differences in e. g. fixation, but from tissue reaction on irradiation (like tumor shrinkage). The extent of these reactions is larger for longer observation times because of time needed for tissue response.

Changes in intertransponder distances suggest geometry variations in the surrounding tissue, possibly modifying the tumor and organs at risk (OAR) geometry in general and especially the relationship of transponder-centroid and tumor-centroid from the planning CT. Interfractional marker-to-tumor-centroid displacements for bronchoscopically implanted gold coils, measured in weekly 4D CBCTs over 7 weeks in 7 patients [20], resulted e. g. in a mean 3D distance change of 5 mm ± 3 mm. A recent study on 71 conventional fractionated lung cancer patients without markers receiving at least a weekly kV CBCT shows a replanning necessity in 60% of the patients due to geometrical changes in tumor or OARs [21].

The Calypso® system measures transponder distances during the localization procedure of each fraction. This data can be used to establish geometrical changes within irradiated lung tissue and may be useful as a future surrogate indicating lung toxicity or therapy response.

## Conclusions

Electromagnetic real-time motion monitoring of the transponder-centroid was feasible for all patients. Detected motion shows that tumor midline motion can be of the same size as the breathing amplitude. In addition, there can be large changes between adjacent time intervals. These results should be kept in mind when choosing treatment technique, proper margins and tracking method, if available. Image-based geometry monitoring should be performed on a regular basis to enable an adaption of treatment to changed geometry of transponders, tumor and OARs.

## Caption Electronic Supplementary Material

Equations for calculation of intra- and interfractional midline variation
