# Supplementary material for: Motion monitoring during a course of lung radiotherapy with anchored electromagnetic transponders: Quantification of inter- and intrafraction motion and variability of relative transponder positions
Source: Strahlenther Onkol. 2017 Jul 21;193(10):840–7. doi: 10.1007/s00066-017-1183-0 (PMC5614910; doi:10.1007/s00066-017-1183-0)
Supplement: Supplementary file 1 — Equations for calculation of intra- and interfractional midline variation [file 66_2017_1183_MOESM1_ESM.pdf]

Strahlentherapie und Onkologie

Supplemetary material to:
'Motion monitoring during a course of lung radiotherapy with anchored electromagnetic transponders: Quantification of inter- and intrafraction motion and variability of relative transponder positions'

Daniela Schmitt*, Simeon Nill, Falk Roeder, Daniela Gompelmann, Felix Herth, Uwe Oelfke,

*Division of Medical Physics in Radiation Oncology, German Cancer Research Center (DKFZ), Heidelberg, Germany and National Center for Radiation Research in Oncology (NCRO), Heidelberg Institute for Radiation Oncology (HIRO)

Now at Department of Radiation Oncology, Heidelberg University Hospital, Heidelberg, Germany
e-mail: Daniela.Schmitt@med.uni-heidelberg.de

Equations for calculation of intra- and interfractional midline variation (norm of 3D values):

With the averaging operator $\langle \ldots \rangle$ and the standard deviation $\sqrt{\text{var}(\ldots)}$.

Midline for point in time t of fraction f: $M_t^f = \frac{1}{101} \sum_{i=t-50}^{t+50} r_i^f$,

defined through a sliding mean of positions r_i^f from 5 s before to 5 s after t.

Initial midline of fraction f with radiation start time t_s: $M_{\text{initial}}^f = M_{t=t_s}^f$.

Intrafractional midline for point in time t of fraction f: $M_{\text{intra},\,t}^f = M_t^f - M_{\text{initial}}^f$.

Mean and SD of the intrafractional midline of fraction f:

$$M_{\text{mean}}^f = \left\langle M_{\text{intra},\,t}^f \right\rangle \quad \text{and} \quad M_{\text{SD}}^f = \sqrt{\text{var}(M_{\text{intra},\,t}^f)}.$$

Interfractional mean and SD of the fraction means per patient:

$$M_{\text{mean}}^{\text{mean}} = \left\langle M_{\text{mean}}^f \right\rangle \quad \text{and} \quad M_{\text{mean}}^{\text{SD}} = \sqrt{\text{var}(M_{\text{mean}}^f)}.$$

Interfractional mean and SD of the fraction SDs per patient:

$$M_{\text{SD}}^{\text{mean}} = \left\langle M_{\text{SD}}^f \right\rangle \quad \text{and} \quad M_{\text{SD}}^{\text{SD}} = \sqrt{\text{var}(M_{\text{SD}}^f)}.$$
